# Jasmonate-Sensitivity-Assisted Screening and Characterization of Nicotine Synthetic Mutants from Activation-Tagged Population of Tobacco (*Nicotiana tabacum* L.)

**DOI:** 10.3389/fpls.2017.00157

**Published:** 2017-02-13

**Authors:** Guoying Yin, Wenjing Wang, Haixia Niu, Yongqiang Ding, Dingyu Zhang, Jie Zhang, Guanshan Liu, Sangen Wang, Hongbo Zhang

**Affiliations:** ^1^College of Agronomy and Biotechnology, Southwest UniversityChongqing, China; ^2^Tobacco Research Institute, Chinese Academy of Agricultural SciencesQingdao, China

**Keywords:** tobacco, activation-tagged population, nicotine, mutant, jasmonate-sensitivity

## Abstract

Nicotine is a secondary metabolite that is important to the defense system and commercial quality of tobacco (*Nicotiana tabacum* L.). Jasmonate and its derivatives (JAs) are phytohormone regulators of nicotine formation; however, the underlying molecular mechanism of this process remains largely unclear. Owing to the amphitetraploid origin of *N. tabacum*, research on screening and identification of nicotine-synthetic mutants is relatively scarce. Here, we describe a method based on JA-sensitivity for screening nicotine mutants from an activation-tagged population of tobacco. In this approach, the mutants were first screened for abnormal JA responses in seed germination and root elongation, and then the levels of nicotine synthesis and expression of nicotine synthetic genes in the mutants with altered JA-response were measured to determine the nicotine-synthetic mutants. We successfully obtained five mutants that maintained stable nicotine contents and JA responses for three generations. This method is simple, effective and low-cost, and the finding of transcriptional changes of nicotine synthetic genes in the mutants shows potentials for identifying novel regulators involved in JA-regulated nicotine biosynthesis.

## Introduction

Mutants play important roles in the cloning of functional genes in crops. Insertional mutagenesis by T-DNA or transposable elements has become a powerful approach to create mutant populations for model plant systems (Parinov and Sundaresan, 2000; Marsch-Martinez et al., 2002). Activation tagging is a T-DNA-based strategy that generates gain-of-function mutations for the research of plant functional genomics (Gou and Li, 2012). The activation-tagging vectors routinely contain a tetramer of the enhancer element from the cauliflower mosaic virus (CaMV) 35S promoter, and the insertion of activation-tag-containing T-DNA causes enhanced expression of the flanking genes (Weigel et al., 2000; Ayliffe and Pryor, 2007). It has been successfully applied in *Arabidopsis thaliana* (Marsch-Martinez et al., 2002), rice (Jeong et al., 2002), tomato (Mathews et al., 2003), legume (Imaizumi et al., 2005) and other plants, but its application in tobacco is much limited.

Tobacco alkaloids are crucial secondary metabolites and defensive compounds that are widely distributed throughout the Solanaceae family (Steppuhn et al., 2004). In cultivated tobacco the predominant alkaloid is nicotine, which accounts for about 90% of the weight of the total alkaloid pool (Saitoh et al., 1985). Nicotine is synthesized in young root tips of tobacco (Dawson, 1941), and subsequently transported to leaves and other tissues through the vascular system (Tso and Jeffrey, 1957; Thurston et al., 1966; Li et al., 2007). Nicotine synthesis in tobacco is inducible by decapitation or jasmonate (JA) treatment, which could induce the activity of nicotine biosynthesis enzymes and promote nicotine production in roots (Rhodes et al., 1989; Hibi et al., 1994; Baldwin, 1996; Howe and Jander, 2008; Shoji et al., 2009). Therefore, these treatments could increase the nicotine content in tobacco leaves (Saunders and Bush, 1979; Baldwin, 1996; Shoji et al., 2008).

Intensive studies suggest that JA-signaling pathway displays an important role in regulating the genes of nicotine biosynthesis enzymes, including quinolinate phosphoribosyltransferase (QPT), ornithine decarboxylase (ODC), putrescine N-methyltransferase (PMT), and isoflavone reductase-like protein (A622; Hibi et al., 1994; Shoji et al., 2000; Voelckel et al., 2001; Thines et al., 2007; Deboer et al., 2009; Memelink, 2009; Sierro et al., 2013; Wang and Bennetzen, 2015). Recently, great progresses have been made in the understanding of the regulatory mechanism underlying nicotine biosynthesis in tobacco. The key JA-signaling regulators CORONATINE INSENSTIVE1 (COI1), Jasmonate ZIM-domain (JAZ) protein, bHLH transcription factor MYC2 and several ethylene response factors (ERFs) have been demonstrated to regulate nicotine synthesis (Shoji et al., 2008; Sheard et al., 2010; Shoji and Hashimoto, 2011, 2014; Sears et al., 2014). A couple of the recently identified nicotine synthetic enzymes, such as BBL (berberine bridge enzyme-like), MATE (multidrug and toxic compound extrusion), NUP1 (nicotine uptake permease), were also shown to be regulated by the JA-signaling pathway (Morita et al., 2009; Hildreth et al., 2011; Kajikawa et al., 2011; Shoji and Hashimoto, 2013; Kato et al., 2014; Shitan et al., 2014, 2015; Lewis et al., 2015). However, the molecular regulatory mechanisms of nicotine biosynthesis are still unclear, and many unknown regulators are yet to be identified through mutant screening or functional genomics.

On the other hand, JA is a phytohormone that regulates seed germination and root elongation in plants (Meyer et al., 1984; Staswick et al., 1992; Farmer et al., 2003; Yoneyama et al., 2014). These phenomena have potentials to be used for identification of regulators involved in the regulation of JA responses and nicotine synthesis. In this study, we developed a novel efficient method to obtain nicotine mutants from activation-tagged tobacco population with the JA-sensitivity assistance. We first screened out mutants that showed abnormal germination time and root length under JA selection pressure. Next, we selected nicotine synthetic mutants from the mutants with abnormal JA responses by measuring their nicotine contents. Finally, we screened out the mutants that maintained stable nicotine contents and JA-sensitivity in three generations. This nicotine mutant screening approach is highly effective, and the analyses showed that the expression levels of key nicotine biosynthetic genes in these mutants were in accordance with the altered JA responses. Thus, this method has the potential to find new nicotine synthetic genes that are mediated by JA-signaling pathway.

## Materials and methods

### Plant materials

The T1 tobacco seeds of 3000 accessions from an activation-tagged population were obtained from the Chinese Tobacco Mutant Library Information Resource (www.tobaccomdb.com) which is supported by Tobacco Research Institute of Chinese Academy of Agricultural Sciences. The seeds were harvested from different T0 plants that were generated by transformation of tobacco cultivar “Honghuadajinyuan” (HD) with the activation-tagging T-DNA vector pSKI015 (Liu et al., 2014). Wild type cultivar HD was used as control for the mutant screening and physiological assays in this study.

### JA-sensitivity assistant screening of tobacco mutants

We randomly picked 100 seeds from each line and divided them into two portions: half of the seeds were germinated in petri dishes containing two layers of filter paper soaked in 10 μM MeJA (Methyl jasmonate; Sigma-Aldrich) in distilled water as the experimental group, while the other half were germinated in petri dishes with filter papers soaked with distilled water as the control group. Hundred seeds of wild-type HD were sown as control in the same way. The seed germination status was daily checked and recorded in the first 1 week. We selected plants that showed obviously longer/shorter germination time than wild-type HD at the presence of JA but no significant difference to the wild-type control at the absence of JA. For those couldn't germinate in 2 weeks, new seeds were sown in petri dish with distilled water to determine whether the seeds were alive. If so, the corresponding lines were considered JA-sensitive.

Another experimental set of seeds were divided into two portions for root elongation assay. Firstly, all the seeds were germinated in petri dishes with water-soaked filter papers for 4 days to let the radicals emerged, and then in half seed set the water was replaced with a solution of 10 μM MeJA. The plants were continuously grown for one more week to measure the root length of each line. We selected plants that showed obviously longer/shorter root length than wild-type HD at the presence of JA but no significant difference to the wild-type control at the absence of JA.

After above experiments, the tobacco lines showing similar JA-sensitivity in both germination and root elongation experiment were selected for further studies.

### Measurement of nicotine content of the mutants

Twenty plants of each mutant line with altered JA-sensitivity were grown in the green house. After 4 months growth, leaves from the middle of plants were collected for nicotine measurement. The plants were then decapitated. After two more weeks growth, leaves from the middle of plants were collected again for nicotine measurement. The leaf nicotine content was determined with a modified UV spectrophotometry method (Willits et al., 1950; Shi et al., 2006). Briefly, 0.15 g tobacco leaves were ground into powder after oven-dried at 60°C, extracted with 18.5% hydrochloric acid for 3 min at room temperature. After centrifugation, the supernatant was transferred into a new tubes with appropriate amount of activated carbon, vortexed for 10 s and then centrifuged. Absorbances of the supernatant were measured at 236 nm (A236), 259 nm (A259), and 282 nm (A282) respectively. The nicotine content (in percentage) was calculated with the formula: 100 × V × 1.059 × [A259 − 0.5 × (A236 + A282)]/34.3/W, where V is the total volume of nicotine extract, 1.059 is the compensation coefficient, 34.3 is the specific extinction coefficient, and W is the weight of sample. This method was compared with the GC/MS (gas chromatography–mass spectrometry) assay and could give similar results as the GC/MS method, thus it was employed for the nicotine measurement due to its high-throughout and low-cost.

Plants with obvious higher and lower nicotine content levels than the control HD plants were selected to generate T2 and T3 progenies. And, the T2 and T3 were grown and tested for JA-sensitivity and nicotine content as described above. Mutants retaining stable JA responses and nicotine contents in three generations were selected as nicotine mutants.

### Classification of the JA-sensitivity and nicotine synthetic levels of the activation-tagged mutants

Taking the characteristics of wild-type HD as control, we classified the JA-sensitivity of the mutant pool into five grades: insensitivity, low sensitivity, normal, sensitivity, and high sensitivity (Table 1), based on the traits of the most sensitive and insensitive mutants of the total population. Analogously, taking the nicotine contents of wild-type HD before and after topping as control, the nicotine contents of mutant pool were classified into five grades: low nicotine, decreased nicotine, normal, increased nicotine, and high nicotine (Table 2), based on the highest and lowest nicotine levels of the mutants of the total population before and after topping.

**Table 1:** **Phenotypic classification of JA response**.

**Material**	**JA-sensitivity**	**Germination time (days)**	**Root length (cm)**
HD	Standard control	5	0.5 ± 0.06
Mutants	Insensitivity	≤3	≥1.01
	Low sensitivity	4	0.61–1.00
	Normal	5	0.41–0.60
	Sensitivity	6–8	0.21–0.40
	High sensitivity	≥9	≤0.20

**Table 2:** **Phenotypic classification of nicotine content**.

**Material**	**Nicotine content**	**Before topping (mg/g FW)**	**After topping (mg/g FW)**
HD	Standard control	0.42 ± 0.09	1.53 ± 0.12
Mutants	Low nicotine	0.00–0.20	0.0–0.60
	Decreased nicotine	0.20–0.35	0.61–1.00
	Normal	0.36–0.50	1.01–2.00
	Increased nicotine	0.51–0.70	2.01–4.00
	High nicotine	≥0.71	≥4.01

### Sample preparation for gene expression analysis

Fifty seeds of the T3 line of each mutant were germinated on sterile plates with 1/2 MS (Murashige and Skoog) medium containing 0.8% agar for 10 days in an indoor growth room. Then, the young seedlings were transferred into a cuboid plastic bottle containing 200 mL liquid 1/2 MS medium, supported by floating plastic plates with Ø 2 mm holes. Four to five seedlings were grown in each bottle. And, the medium was replaced with fresh medium weekly. After 6 weeks growth, the tobacco plants of each line were divided into two groups. Half of the plants were treated with 10 μM MeJA in 1/2 MS medium for 24 h, while the other half were treated with MeJA-free medium as control. Then, the leaves were harvested for genomic DNA extraction and the roots were collected for RNA preparation. The genomic DNA was extracted with CTAB method as previously described by Clarke (2009) and used as template to amplify the *Bar* gene fragment with primer 5′-GCACGGTCAACTTCCGTAC-3′ and 5′-AACCCACGTCATGCCAGTTC-3′ for transgene identification. Samples from the true transgene plants were retained for further experiments.

### Southern blot analysis

50 μg genomic DNA was digested with 50 U of the restriction endonuclease *Eco*RI (TransGen, China). The digested DNA samples were separated on 1% (w/v) agarose gels, and transferred onto Hybond-N^+^ nylon membranes as previously described by Zhang et al. (2012). We amplified the specific sequence of the *Bar* gene using primer 5′-GCTCGGTGTCGTAGATA-3′ and 5′-ACAAGCACGGTCAACGGTCAA-3′ from the binary vector pSKI015 (Liu et al., 2014), and the amplification products were used as DNA probe for Southern blot. The DNA probes labeling and Southern hybridization were performed with reagents from DIG High Prime DNA Labeling and Detection Sarter Kit I (Roche Basel, Switzerland) according to the manufacturer's introduction.

### qRT-PCR analysis

Total RNAs were isolated using Trizol reagent (Invitrogen, USA). All residual DNA was removed by treatment with DNase I. First-strand cDNA was synthesized using PrimeScript 1st Strand cDNA Synthesis Kit (Takara, Japan). The qRT-PCR was performed under the following conditions: predenaturation at 95°C for 5 min; then 40 cycles of denaturation at 95°C for 20 s, annealing at 58°C for 20 s, and extension at 72°C for 40 s. Each reaction had three technical replicates. Followings are primers used for qRT-PCR: 5′-CCACACAGGTGTGATGGTTG-3′ and 5′-GTGGCTAACACCATCACCAG-3′ for *NtActin*; 5′-TCACCCATTTCTCTCTCTCTCTC-3′ and 5′-GAGGTAACAGCAGCAGTAGTAG-3′ for *NtMYC2*; 5′-TTGATCAATGGGAGGTTTGA-3′ and 5′-TCAGGGCACCACTAGAAATG-3′ for *NtQPT*; 5′-GTTTCCGACGACTGTGTTTG-3′ and 5′-ATTGGACCCAGCAGCTTTAG-3′ for *NtODC*; 5′-AAAATGGCACTTCTGAACAC-3′ and 5′-CCAGGCTTAATAGAGTTGGA-3′ for *NtPMT*; 5′-GATGGAAATCCCAAAGCAAT-3′ and 5′-GCAGGTGGTCTCATGTGAAG-3′ for *NtA622*; 5′-TTGGTTACAAACGGGTGGAT-3′ and 5′-AAAATTCGGGGTGTCATCAA-3′ for *NtNUP1*; 5′-GATGGACGGTTATTAGACCG-3′ and 5′-ATTTTCCAGGCATAAACAATG-3′ for *NtBBL* genes. The relative expression levels were calculated based on the threshold cycles of interested genes and that of internal control using the equation 2^(−ΔΔCT)^ as described by Zhang et al. (2012).

### Gene accessions

*NtActin* (X63603), *NtMYC2* (HM466974), *NtQPT* (AJ748262), *NtODC* (AF127242), *NtPMT* (AF280402), *NtA622* (D28505), *NtBBLs* (AB604219, AM851017, AB604220, AB604221), *NtNUP1* (GU174267).

## Results

### Screening of nicotine-synthetic mutants from activation-tagged population

Of the 3000 T1 transgenic lines that were screened with 10 μM MeJA, over 90% showed a JA response similar to that of the wild-type control. We identified 48 lines with abnormal JA responses in both germination and root elongation: 20 JA-insensitive lines and 28 JA-sensitive lines. Of the 20 JA-insensitive lines, three were classed as “insensitivity” and exhibited high JA insensitivity, accounting for 0.10% of the total T1 lines. The other 17 lines were classed as “low sensitivity.” Of the 28 JA-sensitive lines, four were highly JA sensitive and were classed as “high sensitivity,” accounting for 0.13% of the total T1 population. The other 24 lines were classed as “sensitivity” (Table 3). We retained the 48 abnormal JA response lines and grew them in a greenhouse for nicotine-content determination.

**Table 3:** **Statistics of T1 lines showing altered JA-sensitivity**.

**Classification**	**Germination**		**Root elongation**		**Lines of similar change in both tests**	
	**Number**	**Percentage of T1 lines (%)**	**Number**	**Percentage of T1 lines (%)**	**Number**	**Percentage of T1 lines (%)**
Insensitivity	24	0.80	32	1.07	3	0.10
Low sensitivity	75	2.50	87	2.90	17	0.57
Normal	2,832	94.40	2,774	92.47	2,011	67.03
Sensitivity	51	1.70	94	3.13	24	0.80
High sensitivity	18	0.60	13	0.43	4	0.13

Of the 48 lines with abnormal JA response, 10 showed similar nicotine-content changes before and after topping. Among these 10 lines, 6 had decreased nicotine content, and 4 had increased nicotine content. In the 6 nicotine decreased lines, 2 had extra-low nicotine and were classed as “low nicotine” mutants, and the other 4 lines were classed as “decreased nicotine,” accounting for 0.20% of the total T1 lines. Of the 4 nicotine increased lines, 2 had extra-high nicotine and were classed as “high nicotine,” and the other two lines were classed as “increased nicotine,” accounting for 0.13% of the total T1 lines (Table 4).

**Table 4:** **Nicotine contents of T1 lines showing consistent JA-sensitivity in both germination and rooting assays**.

**Classification**	**Before topping change**		**After topping change**		**Lines of similar change in both tests**	
	**Number**	**Percentage of T1 lines (%)**	**Number**	**Percentage of T1 lines (%)**	**Number**	**Percentage of T1 lines (%)**
Low nicotine	5	0.17	4	0.13	2	0.07
Decreased nicotine	6	0.20	7	0.23	4	0.13
Normal	25	0.83	22	0.73	16	0.53
Increased nicotine	8	0.27	9	0.30	2	0.07
High nicotine	4	0.13	6	0.20	2	0.07

The accession numbers of the 10 identified nicotine mutants are 11MHT1009492, 11MHT1009915, 11MHT1009979, 11MHT1009785, 11MHT1009410, 11MHT1009060, 11MHT1010449, 11MHT1010579, 11MHT1009196, and 11MHT1009936, and will be referred to as *nit1, nit2, nit3, nit4, nit5, nit6, nit7, nit8, nit9*, and *nit10* (numbered in an order of nicotine content increase). These mutants showed similar trends in changes of both JA response and nicotine synthesis: a reduced JA response was accompanied by decreased nicotine content and vice versa (Table 5).

**Table 5:** **The correlation between JA response and nicotine content in mutants**.

**Material**	**Accession**	**JA response**	**Nicotine content**	**Consistency**
HD	— —	Standard control	Standard control	–
*nit1*	11MHT1009492	Insensitivity	Low nicotine	Yes
*nit2*	11MHT1009915	Insensitivity	Low nicotine	Yes
*nit3*	11MHT1009979	Low sensitivity	Decreased nicotine	Yes
*nit4*	11MHT1009785	Low sensitivity	Decreased nicotine	Yes
*nit5*	11MHT1009410	Low sensitivity	Decreased nicotine	Yes
*nit6*	11MHT1009060	Low sensitivity	Decreased nicotine	Yes
*nit7*	11MHT1010449	Sensitivity	Increased nicotine	Yes
*nit8*	11MHT1010579	High sensitivity	High nicotine	Yes
*nit9*	11MHT1009196	High sensitivity	High nicotine	Yes
*nit10*	11MHT1009936	High sensitivity	High nicotine	Yes

“*Consistency” indicates the JA response and nicotine content of the material showing similar change trends*.

### Verification of the nicotine synthetic mutants

**Figure 1:**
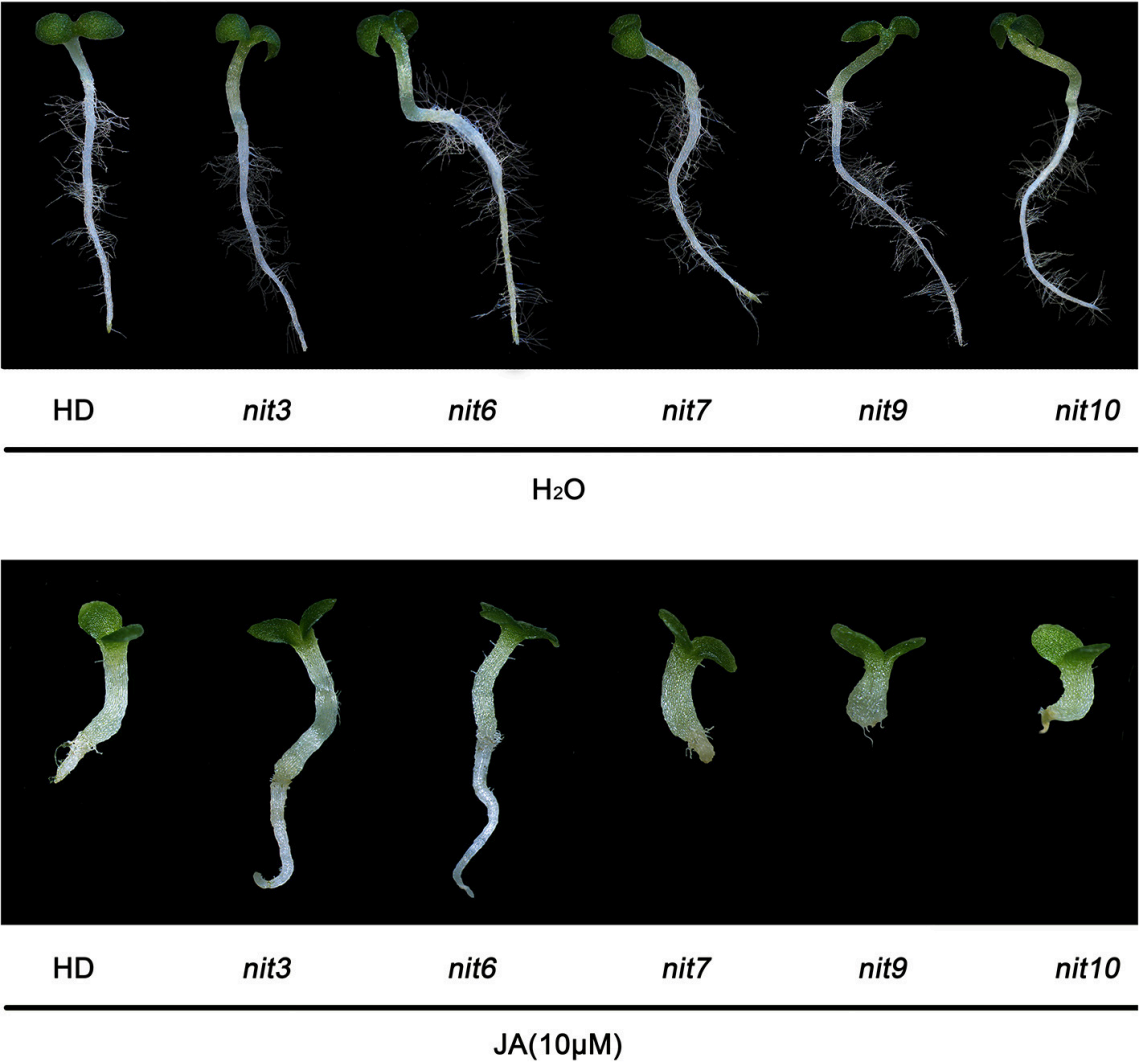
**Root elongation of the verified nicotine synthetic mutants upon JA treatment**. Plants from left to right are HD (control), *nit3* (11MHT1009979), *nit6* (11MHT1009060), *nit7* (11MHT1010449), *nit9* (11MHT1009196), and *nit10* (11MHT1009936), respectively. Top panel is control treatment with distilled water.

We then determined the JA-sensitivity and nicotine contents of the above lines in three generations to verify the stability of these traits during different generations. Five mutants (*nit3, nit6, nit7, nit9, nit10*) were found to exhibit similar JA response and nicotine synthetic levels over three generations. JA-sensitivity assay confirmed *nit3* and *nit6* as “low sensitivity.” These two lines germinated 1 day earlier than control, and their root lengths were 0.762 and 0.755 cm, respectively, which were 0.341 and 0.334 cm longer than that of the control. Nicotine measurement further confirmed *nit3* and *nit6* as “decreased nicotine” and showed low nicotine. Their nicotine contents were 0.212 and 0.213 mg/g, respectively before topping, which were about 50% of that of the control. After topping, their nicotine contents were 0.615 and 0.992 mg/g, which were also less than the control. *nit7* was confirmed as “sensitivity” in JA response and as “increased nicotine” by nicotine measurement. Its nicotine content was 0.512 mg/g before topping and 2.074 mg/g after topping, which were higher than the control under both conditions. JA-sensitivity assay also confirmed *nit9* and *nit10* as “high sensitivity,” which showed greatly enhanced JA-sensitivity. These two lines germinated on the 12th day and the 14th day, respectively, which were over 1 week later than the control. Their root lengths were 0.187 and 0.153 cm, respectively, which were 0.234 and 0.268 cm shorter than that of the control (Table 6; Figure 1). Nicotine measurement showed that *nit9* and *nit10* are “high nicotine” mutants with nicotine contents of 0.701 and 0.811 mg/g before topping and 4.612 and 4.205 mg/g after topping, respectively, showing strikingly higher nicotine contents than that of the control (Table 7).

**Table 6:** **JA responsive characteristics of the verified nicotine synthetic mutants**.

**Material**	**Germination time (d)**	**Root length (cm)**	**JA response**
HD	5	0.421 ± 0.056	Standard control
*nit3*	4	0.762 ± 0.101	Low sensitivity
*nit6*	4	0.755 ± 0.084	Low sensitivity
*nit7*	7	0.314 ± 0.054	Sensitivity
*nit9*	12	0.187 ± 0.018	High sensitivity
*nit10*	14	0.153 ± 0.021	High sensitivity

**Table 7:** **Nicotine contents of the verified nicotine synthetic mutants**.

**Material**	**Before topping (mg/g FW)**	**After topping (mg/g FW)**	**Nicotine content**
HD	0.416 ± 0.098	1.532 ± 0.211	Standard control
*nit3*	0.212 ± 0.042	0.615 ± 0.024	Decreased nicotine
*nit6*	0.213 ± 0.031	0.992 ± 0.029	Decreased nicotine
*nit7*	0.512 ± 0.024	2.074 ± 0.242	Increased nicotine
*nit9*	0.701 ± 0.132	4.612 ± 0.358	High nicotine
*nit10*	0.811 ± 0.154	4.205 ± 0.342	High nicotine

### Molecular characteristics of the nicotine synthetic mutants

**Figure 2:**
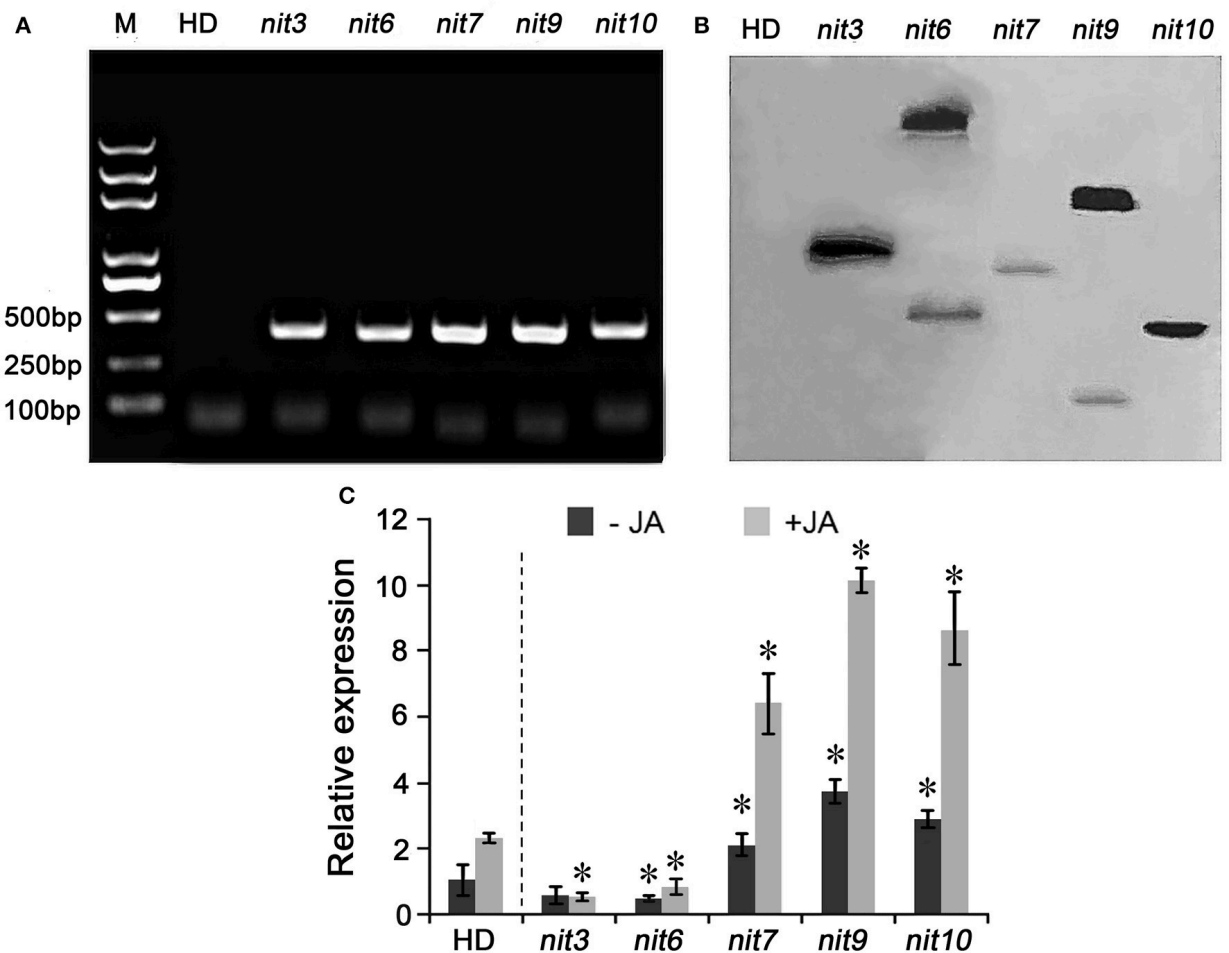
**Molecular characterization of the nicotine synthetic mutants. (A)** PCR amplification of *Bar* gene in nicotine synthetic mutants. M indicate DNA molecular weight markers. **(B)** Southern blot analysis of T-DNA insertion in the nicotine synthetic mutants. **(C)** Expression of *NtMYC2* in the mutant. −JA indicates materials without MeJA treatment; +JA indicates materials treated with MeJA for 24 h. Expression level of *NtMYC2* in untreated wild-type HD was set as “1.” Asterisk indicates significant difference to the transcript level of wild-type HD under the same treatment (*P* < 0.05, Student's *t*-test). Error bar = ±*SD*. HD, wild-type HD; *nit3/6/7/9/10* indicate different mutants.

To confirm the transgenic nature of these mutants, PCR and Southern hybridization analyses were carried out. PCR amplification assays showed that all five mutants tested were positive in holding the *Bar* gene, the herbicide-resistance gene in the activation-tagging vector pSKI015 (Figure 2A). Furthermore, the *Bar* gene was used as a probe for the Southern hybridization, and the results showed that *nit6* and *nit9* had two T-DNA insertions in their genomes, and *nit3, nit7*, and *nit10* had one T-DNA insertion each (Figure 2B).

Then, the expression level of *NtMYC2*, an important regulator of JA-signaling pathway (Zhang et al., 2012), was determined by qRT-PCR assay to characterize molecular mechanism underlying the differential JA sensitivities of the identified mutants. The results turned out that the transcript levels of *NtMYC2* was greatly increased and highly inducible by MeJA in the JA-sensitity mutants *nit7, nit9*, and *nit10*, while its expression levels was dramatically attenuated in the JA-insensitivity mutants *nit3* and *nit6* (Figure 2C). The transcript levels of *NtMYC2* in the other mutants with altered JA-sensitivity (i.e., *nit1, nit2, nit4, nit5, nit8*) were also analyzed by qRT-PCR, and showed a consistency between the JA-sensitivity and *NtMYC2* expression levels (Figure S1). These results provided the molecular information correlated with JA-sensitivity of these identified mutants.

### Expression of nicotine-synthetic genes in the nicotine synthetic mutants

**Figure 3:**
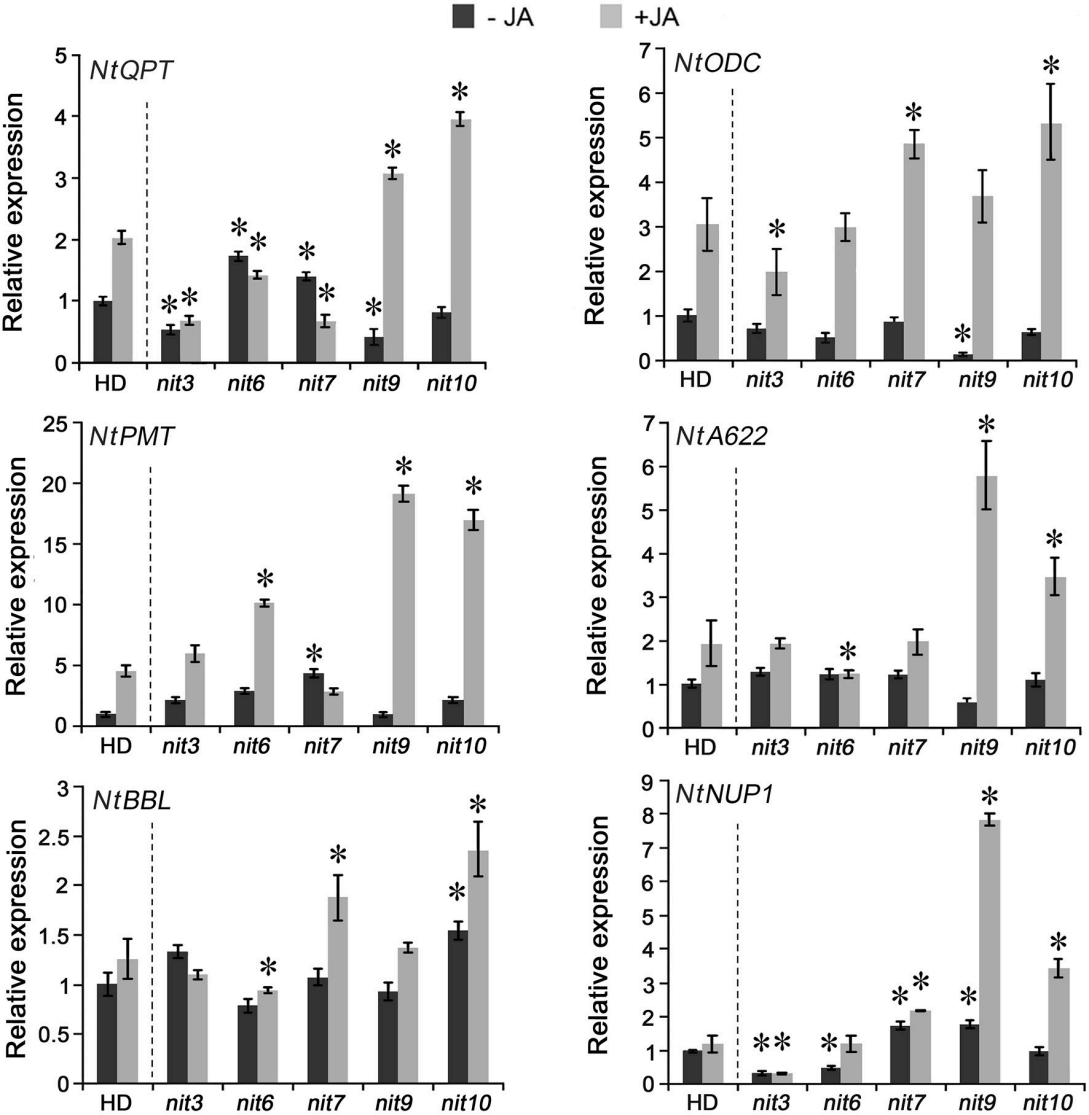
**Expression of nicotine biosynthetic genes in the mutant *nit3/6/7/9/10***. −JA indicates materials untreated with MeJA; +JA indicates materials treated with MeJA for 24 h. Expression level of each gene in untreated wild-type HD was set as “1.” Asterisk indicates significant difference to the transcript level of wild-type HD under the same treatment (*P* < 0.05, Student's *t*-test). *nit3/6/7/9/10* indicate different mutants. Error bar = ±*SD*.

To reveal the molecular basis of the mutants with altered nicotine synthesis, we analyzed the gene expression levels of nicotine biosynthetic enzymes, including NtQPT, NtPMT, NtODC, NtA622, NtBBL, and NtNUP1, in the roots with or without MeJA treatment. The results indicated that the expression levels of these genes showed significant differences among the mutants (Figure 3). *nit3* showed lower relative gene expression levels of *NtQPT* and *NtNUP1* in the absence of MeJA, and exhibited lower expression levels of *NtQPT* and *NtODC* after MeJA treatment. The decreased-nicotine mutant *nit6* showed lower gene expression levels of *NtNUP1* without MeJA treatment, and was found no increase in *NtQPT* and *NtA622* following MeJA treatment, while the expression level of *NtPMT* promoted by MeJA treatment. The expression levels of genes encoding NtQPT, NtPMT and NtNUP1 were higher in *nit7* than those in the control before MeJA treatment, and the expression levels of *NtODC, NtBBL*, and *NtNUP1* were significantly increased after MeJA treatment. In the absence of MeJA, *NtNUP1* showed higher level of expression in the high nicotine mutant *nit9*, whereas *NtQPT* and *NtODC* showed lower levels. After application of MeJA, *NtQPT, NtPMT, NtA622*, and *NtNUP1* showed strikingly higher levels of expression in *nit9* compared with the control. The high nicotine mutant *nit10* had higher level of expression of *NtBBL* in the absence of MeJA, and showed strongly induced expression of *NtQPT, NtODC, NtPMT, NtA622, NtBBL*, and *NtNUP1* by MeJA treatment. These results suggested that the expression levels of nicotine synthetic genes are variably affected by JA treatment in these nicotine mutants, consistent with the roles of JA in inducing nicotine formation. We also analyzed the expression of above genes in the mutant *nit1/2/4/5/8*, which showed no significant changes in nicotine levels, and found less obvious differences to that of the control (Figure S2).

## Discussion

### JA-sensitivity assisted screening of nicotine mutants is a novel and effective method for tobacco

Obtaining mutants through phenotypic analysis under selection pressure is a common method for mutant library screening. It helps the selection of desirable mutants and is widely used in plants. To date, many resistant and sensitive mutants have been screened out in this way (Harlman, 1984; Ishida and Kumashiro, 1988; Saleki et al., 1993; Feys et al., 1994; He et al., 2009; Zha et al., 2015). Nevertheless, selection pressure for screening can only identify mutants that show abnormal responses to selection conditions such as resistance to salt, acidity, and disease (Ishida and Kumashiro, 1988; Saleki et al., 1993; He et al., 2009). JA plays pivotal roles in plant defense responses. In recent years, the finding of root growth sensitivity to JA has allowed scientists to identify many JA-insensitive mutants for studies in JA-signaling pathway (Zha et al., 2015). Although nicotine synthesis in tobacco is correlated with root development (Staswick et al., 1992; Shitan et al., 2014), there were no reports about identification of nicotine mutants based on JA-sensitivity.

Nicotine is synthesized in the roots of tobacco and regulated by the phytohormone JA (Meyer et al., 1984; Staswick et al., 1992; Shoji et al., 2000). Adoption of the physiological phenomenon of JA-regulated root development to the selection of nicotine synthetic mutant has a potential to identify regulator involved in both JA response and nicotine synthesis. And, this approach could also be applied to high-throughout screening of nicotine mutants from mutant pools. The JA-assisted screening of nicotine mutant was based on determining the JA-sensitivity of both germination and root elongation of the mutants. Using 10 μM MeJA as selection pressure, we initially screened out 48 mutants that exhibited abnormal JA-sensitivity during germination and root elongation from 3000 T1 lines. Then we selected 10 nicotine mutants from the 48 lines by determining their nicotine contents before and after topping. The results implied a positive association between JA-sensitivity and nicotine content among the 10 mutants.

The JA selection pressure was able to eliminate 2952 T1 lines (more than 98.4%) at germination and seedling stages, therefore we were therefore able to grow the 48 remaining lines in the greenhouse, which is helpful to save time and cultivation space. By further screening, we obtained 10 nicotine mutants from the 48 lines, and five nicotine mutants could maintain stable nicotine contents during three generations. These mutants also showed stable JA-sensitivity during three generations. In addition, the expression of genes for enzymes involved in nicotine biosynthesis in the nicotine mutants was also affected by JA, indicating that the nicotine biosynthesis of these mutants is regulated by the JA signal pathway. Mutants selected in this way are of values to dissect the mechanism underlying JA-mediated nicotine biosynthesis.

### Correlation between the changes of JA response and nicotine content

**Figure 4:**
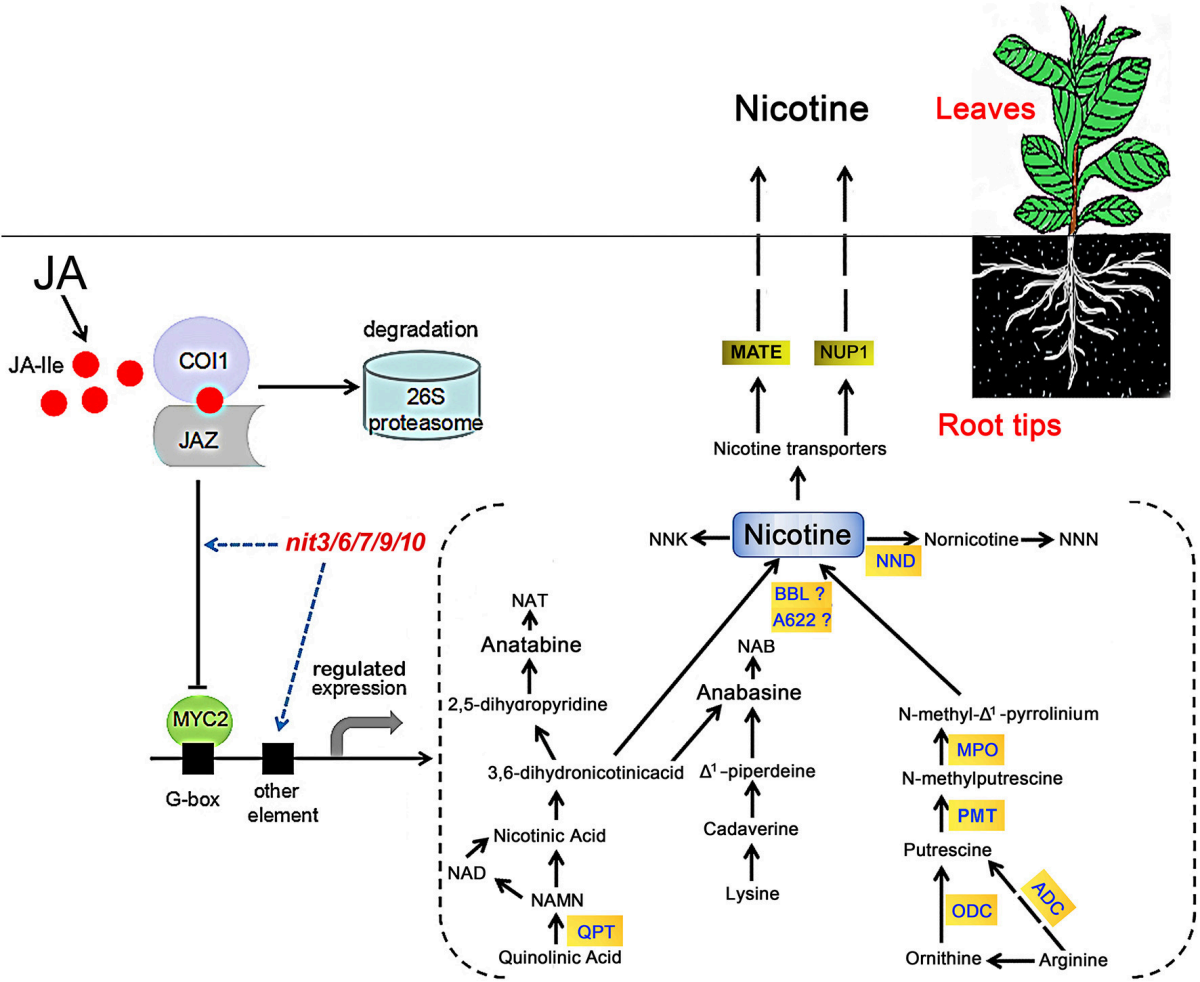
**Model of JA-stimulated induction of nicotine biosynthesis in tobacco**. In the presence of JA, COI1 binds to JAZ and mediates its degradation through the 26S proteasome complex. After the removal of JA pathway suppressor JAZ protein, MYC2 transcription factor is released to bind the G-box motif, resulting in the activation of nicotine biosynthetic genes. The *nit* mutants may alter nicotine synthesis via modifying tobacco JA responses or directly regulating the genes involved in nicotine synthesis. The model for nicotine synthetic pathway is based on the reviews by Dewey and Xie (2013) and by Lewis et al. (2015). NNK, 4-(methylnitrosamino)-1-(3-pyridyl)-1-butanone; NND, nicotine N-demethylase; NNN, N-nitrosonornicotine; NAT, N-nitrosoanatabine; NAB, N-nitrosoanabasine; ADC, arginine decarboxylase.

Nicotine is composed of a piperidine ring and a pyrrolidine ring, derived from the pyridine nucleotide cycle and the methylpyrroline pathway respectively (Tiburcio and Galston, 1986; Wagner et al., 1986; Dewey and Xie, 2013). In the pyridine nucleotide cyclic pathway, quinolinic acid is converted to nicotinic acid mononucleotide (NAMN) by QPT, which can enter into the pyridine nucleotide cycle to form nicotinic acid (Dewey and Xie, 2013; Sierro et al., 2013; Wang and Bennetzen, 2015). Subsequently, nicotinic acid forms nicotine through a multi-step process. The methylpyrroline pathway originates with ornithine directly, which is decarboxylated by ODC to form putrescine (Baldwin, 1998; Shoji et al., 2008), putrescine is converted to N-methylputrescine through the action of methylation by PMT (Shoji et al., 2000; Voelckel et al., 2001). After the deamination of N-methylputrescine by myeloperoxidase (MPO), the product N-methylaminobutanal can spontaneously cyclize to form N-methyl-Δ1-Pyrrolinium, which is directly involved in the formation of nicotine (Chou and Kutchan, 1998; Katoh et al., 2007). The nicotine biosynthesis enzymes isoflavone reductase-like (A622; Deboer et al., 2009) and BBL catalyze the final step in the formation of nicotine from the piperidine and pyrrolidine rings (Kajikawa et al., 2011; Lewis et al., 2015). Then, the synthesized nicotine was uptaken to tobacco leaves by nicotine transporters MATE and NUP1 (Figure 4). It has been noted that phytohormone levels are important in nicotine biosynthesis in tobacco (Shoji et al., 2008). Jasmonic acid and its derivatives, collectively called jasmonate (JA), are the most crucial cellular phytohormone regulators of nicotine biosynthesis in tobacco (Dewey and Xie, 2013; Wang and Bennetzen, 2015). JA-induced nicotine formation is mediated by CORONATINE INSENSTIVE1 (COI1), JAZ, and bHLH/MYC2 genes in tobacco roots (Thines et al., 2007; Shoji et al., 2008; Sheard et al., 2010; Todd et al., 2010). Applying exogenous JA, or increasing endogenous levels of JA, could both trigger COI1-mediated degradation of ZIM-domain-containing (JAZ) transcriptional repressor, thereby releasing transcription factors including bHLH or MYB families to activate the downstream JA responses (Chung et al., 2008; Zhang et al., 2012; Kazan and Manners, 2013), which includes the genes encoding nicotine biosynthesis enzymes (QPT, ODC, PMT, A622, BBL, NUP1 etc.; Figure 4).

In our study, the identified nicotine mutants exhibited consistent JA responses in both germination and root elongation, furthermore, the transcriptional assays revealed a good consistency between the JA-sensitivity and the expression level of *NtMYC2*, an important regulatory gene in JA-signaling pathway, in these mutants. These data support a close correlation between JA response and nicotine content. The five nicotine mutants showed differential changes in the expression of nicotine biosynthetic genes. *nit3* is an extra-low nicotine mutant and showed high JA insensitivity and presented lower relative gene expression levels of *NtQPT* and *NtNUP1* in the absence of MeJA. The expression of genes encoding NtQPT and NtA622 was not increased in response to MeJA treatment. We predicted that the nicotine content reduction in *nit3* might be related to the enzymatic activity decrease of NtQPT, NtA622, and NtNUP1, which are regulated by JA-signaling pathway, and it might also affect the nicotine formation by reducing the plant sensitivity to JA. Similarly, low nicotine mutant *nit6* showed high JA insensitivity, lower expression level of *NtNUP1* gene without MeJA treatment, and showed no increase in the expression of *NtQPT* and *NtA622* genes following MeJA treatment. However, the expression of *NtPMT* in *nit6* was induced by MeJA treatment. *nit7* presented high nicotine content and JA-sensitivity, and also showed increased expression of *NtODC, NtBBL*, and *NtNUP1* upon MeJA treatment. *nit9* and *nit10* presented extra-high nicotine and high JA-sensitivity. After application of MeJA, the expression levels of most of the tested nicotine biosynthetic genes were strikingly promoted in *nit9* and *nit10*, compared to that of the control. The above information suggested that these mutants possess differential regulation on the nicotine synthetic genes, implying the presence of differential regulatory mechanism underlying the JA response and nicotine accumulation in these mutants (Figure 4).

### Conclusion and perspectives

Although, the nicotine biosynthetic pathway regulated by the JA-signaling pathway has been intensively defined, the underlying molecular mechanism is still unclear. Utilizing the advantages of JA-regulated root responses, this study identified 5 nicotine synthetic mutants from an activation-tagged population and these mutants exhibited consistent changes in JA response and nicotine synthesis levels, supporting a close correlation between JA response and nicotine synthesis. This study also provided a feasible way to identify regulators involved in the regulation of both JA response and nicotine synthesis, which is helpful to reveal the regulation of nicotine synthesis by the JA-signaling pathway.

## Author contributions

GY carried out the experimental work and wrote the manuscript. WW, HN, YD, DZ, and JZ helped in experimental work and data analyses. GL and SW helped in conceiving of the study and revised the manuscript. HZ conceived the study and wrote the manuscript. All authors read and approved the final manuscript.

## Funding

This work was supported by the Science and Technology Innovation Program of Chinese Academy of Agricultural Sciences (Elite youth program to HZ, ASTIP-TRIC05), the Key Special Program of China National Tobacco Corporation [TS-02-20110014; 110201301005 (JY-05)], the Program of Chongqing Tobacco Company (NY20140403030022).

### Conflict of interest statement

The authors declare that the research was conducted in the absence of any commercial or financial relationships that could be construed as a potential conflict of interest.
