# Supplementary material for: Jasmonate-Sensitivity-Assisted Screening and Characterization of Nicotine Synthetic Mutants from Activation-Tagged Population of Tobacco (Nicotiana tabacum L.)
Source: Front Plant Sci. 2017 Feb 13;8:157. doi: 10.3389/fpls.2017.00157 (PMC5303748; doi:10.3389/fpls.2017.00157)
Supplement: Supplementary file 1 [file Image1.PDF]

Supplementary Material:

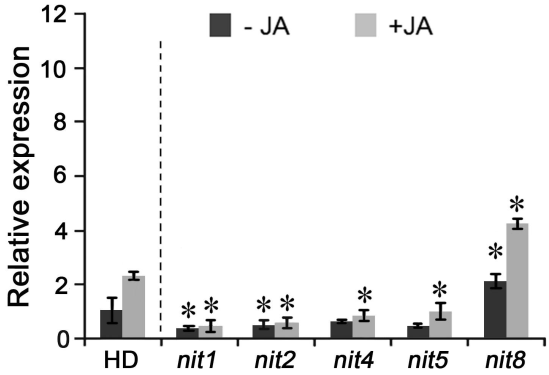

Figure S1. Expression of *NtMYC2* in the mutant *nit1/2/4/5/8*.

–JA indicates materials untreated with MeJA; +JA indicates materials treated with MeJA for 24 h. Expression level of *NtMYC2* in untreated wild-type HD was set as '1'. Asterisk indicates significant difference to the transcript level of wild-type HD under the same treatment (*P*<0.05, Student's *t* test). *nit1/2/4/5/8* indicate different mutants. Error bar=±SD.

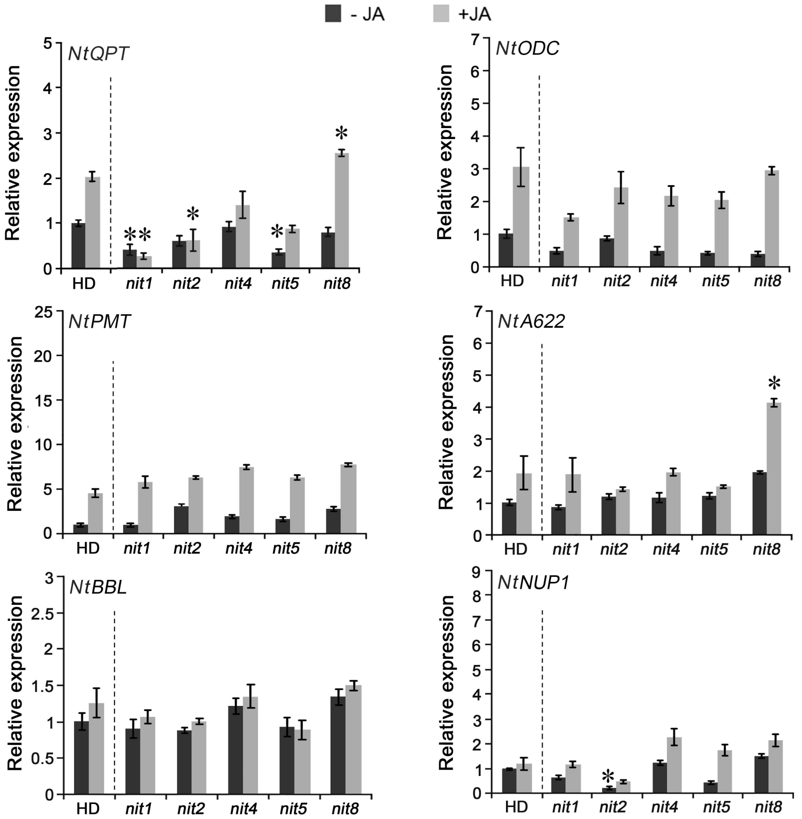

Figure S2. Expression of nicotine biosynthetic genes in the mutant *nit1/2/4/5/8*.

–JA indicates materials untreated with MeJA; +JA indicates materials treated with MeJA for 24 h. Expression level of each gene in untreated wild-type HD was set as '1'. Asterisk indicates significant difference to the transcript level of wild-type HD under the same treatment (*P*<0.05, Student's *t* test). *nit1/2/4/5/8* indicate different mutants. Error bar=±SD.
